# Ex vivo biomechanical comparison of 2.7 mm string-of-pearl plate versus screw/wire/Polymethylmethacrylate composite fixation and 2.7 mm veterinary acetabular plate for repair of simulated canine acetabular fractures

**DOI:** 10.1186/s12917-019-2024-4

**Published:** 2019-08-09

**Authors:** Jonathan A. Blakely, James R. Butler, Lauren B. Priddy, Emily M. McCabe, Javier N. Avendaño, Steve H. Elder, Robert Wills

**Affiliations:** 1 Mississippi State University College of Veterinary Medicine, 240 Wise Center Drive, Mississippi State, MS 39762 USA; 2 Mississippi State University College of Agricultural and Biological Engineering, Box 9632, 130 Creelman Street, Mississippi State, MS 39762 USA

**Keywords:** String-of-pearls, Canine, Acetabulum, Fracture, Biomechanics

## Abstract

**Background:**

Acetabular fractures comprise 12–30% of canine pelvic fractures and require accurate anatomic reduction and rigid stability to ensure proper healing and minimize future osteoarthritis. Many techniques have been used to repair these fractures, with common techniques including veterinary acetabular plates or use of screw/wire/polymethylmethacrylate constructs. String-of-Pearl™ plating systems have also been used clinically but there is a lack of research supporting their use for these fractures. The purpose of this study was to compare fracture reduction accuracy, biomechanical characteristics, and mode of failure between String-of-Pearls™, veterinary acetabular plates, screw/wire/polymethylmethacrylate constructs in a simulated, ex-vivo acetabular fracture model. We hypothesized that the String-of-Pearls™ constructs would have equivalent or greater mechanical properties and reduction compared to the other constructs.

**Results:**

The mean craniocaudal acetabular diameter before fixation (mean 25.2 mm; range 20 mm – 30.1 mm) was not significantly different from after fixation (mean 23.9 mm; range 20 mm – 28.3 mm) for any fixation method. Comparison of reduction scores between groups revealed no significant differences. No significant differences were noted for cyclical displacement or stiffness. There was significant difference with superior failure load of String-of-Pearls™ compared to screw/wire/polymethylmethacrylate in the 75th percentile of animal weight (*P* = 0.0021), and superior failure load of String-of-Pearls™ compared to veterinary acetabular plates in the 50th (*P* = 0.0232) and 75th percentiles (*P* = 0.0058). Stiffness of the String-of-Pearls™ construct was significantly greater than the veterinary acetabular plate construct (*P* = 0.0417). For ultimate load, String-of-Pearls™ constructs were significantly greater than screw/wire/polymethylmethacrylate (*P* = 0.0331) and veterinary acetabular plates (*P* = 0.0218).

**Conclusion:**

Although the ease of application for the String-of-Pearls™ implant was subjectively better than other implants, no significant differences were found in fracture reduction scores. The String-of-Pearls™ constructs were stiffer than veterinary acetabular plates and exhibited greater failure and ultimate loads compared to veterinary acetabular plates and screw/wire/polymethylmethacrylate fixations. The String-of-Pearls™ implant appears to be a suitable fixation choice for simple canine acetabular fractures.

## Background

Acetabular fractures, comprising 12–30% of canine pelvic fractures, require accurate anatomical reduction and rigid stability to encourage primary bone healing and reduce the development of osteoarthritis [1–4]. Repair of acetabular fractures has been reported using small fragment plates, reconstruction plates with or without plate luting, veterinary acetabular plates (VAP), tubular plates, and composite fixation using screws/wire/polymethylmethacrylate (SWP) constructs [5–8]. Plate fixation is generally successful in restoring joint congruity and allowing fracture healing, but reported complications include screw pullout or breakage [2, 9, 10] due to implant size limitations and limited screw purchase caudal to the fracture site [9, 10]. In addition, plate contouring is complicated by the irregular dorsal acetabular rim and can lead to malalignment upon tightening of screws, resulting in osteoarthritis and poor function [2, 6, 7]. To combat malalignment formation, tension-band techniques strengthened by polymethylmethacrylate and plate luting have been reported [1–3, 6–8, 11, 12]. However, potential complications with polymethylmethacrylate fixation include decreased range of motion on abduction, excessive protrusion of the interfragmentary Kirschner wires into the pelvic canal, thermal injury during cement curing, and a difficult-to-remove nidus should bacterial infection occur [3, 6, 8, 11]. The biomechanical strength of SWP fixation under a single load to failure has been previously reported; however, the effects of more realistic cyclical loading on this fixation method has yet to be reported in the veterinary literature [3, 6, 8, 11].

Locking plate technology creates a “toggle-free” fixed angle construct and reduces the need for accurate plate contouring, which is beneficial with the irregular dorsal acetabulum [7, 13, 14]. Additionally, locking plate systems maintain primary and secondary reduction after placement and help preserve periosteal blood supply -- a significant benefit over standard VAP and dynamic compression plates [7, 15]. Locking plates are also beneficial in osteopenic bone or when limited screw purchase is possible, which is advantageous in acetabular fixation where only 2 screws are frequently placed in the caudal fracture segment [5, 2, 7].

String-of-Pearl (SOP) locking plate design allows increased contouring ability as up to 40° bending and 20° twisting [1] between internodes is possible without compromising locking capability. The ease of contouring and application combined with the strength of a locking plate make SOP design an acceptable means of fixation for acetabular fractures. However, the decrease in torsional strength compared to other implants may be a disadvantage as many studies have shown acetabular fixation to fail via ventrolateral rotation of the caudal segment [8, 11]. A retrospective study evaluating the use of SOP plates in acetabular fractures in 3 dogs found the construct to allow appropriate reduction and fixation with no post-operative complications. Acceptable limb function was reported in all 3 cases [1]. To our knowledge, there have been no previous biomechanical evaluations of acetabular fractures repaired using SOP plating systems.

The purpose of this study was to compare fracture reduction accuracy, biomechanical characteristics, and mode of failure between SOP, VAP, SWP constructs in a simulated acetabular fracture model. We hypothesized that the SOP constructs would have equivalent or greater mechanical properties and superior reduction compared to the other constructs.

## Results

Dogs were adult mixed breed and consisted of 7 spayed females, two intact females, three intact males, and 6 neutered males for a total of 36 hemipelves divided into three groups (12 hemipelves per test group). Mean weight of the cadavers was 28 kg (median 27 kg; range 20 kg – 37 kg). The mean craniocaudal acetabular diameter before fixation (mean 25.2 mm; range 20 mm – 30.1 mm) was not significantly different from after fixation (mean 23.9 mm; range 20 mm – 28.3 mm) for any fixation method (*P* = 0.63).

For the acetabular reduction scores, there were 8 Grade I, 3 Grade II, and 1 Grade III for the SWP group; 2 grade I, 8 grade II, and 2 grade III for the SOP group; and 3 grade I, 6 grade II, and 3 grade III for the VAP group. Comparison of reduction scores between groups revealed no significant differences (*P* = 0.1).

**Fig. 1:**
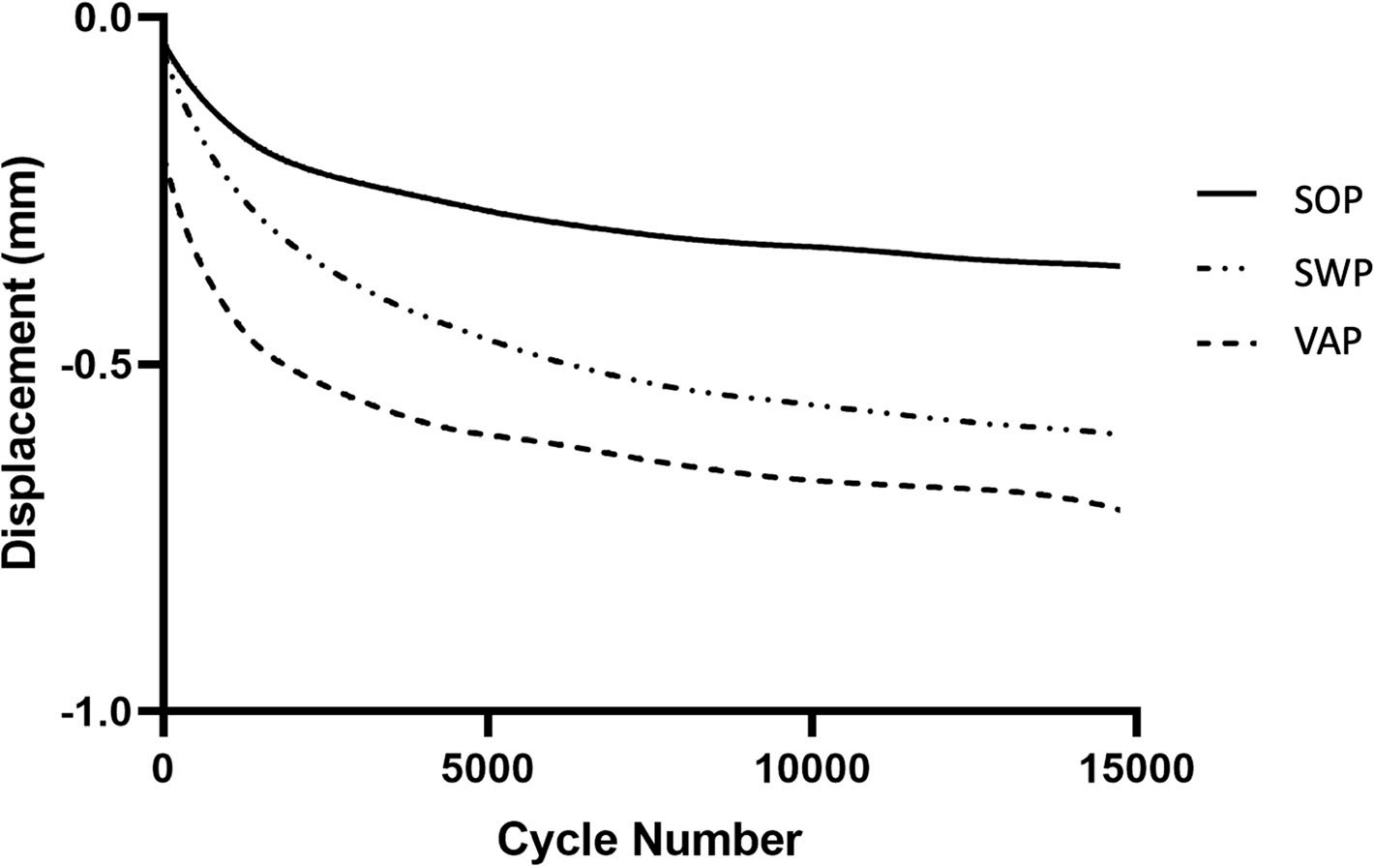
Cyclic displacement for SOP, SWP, and VAP constructs

**Fig. 2:**
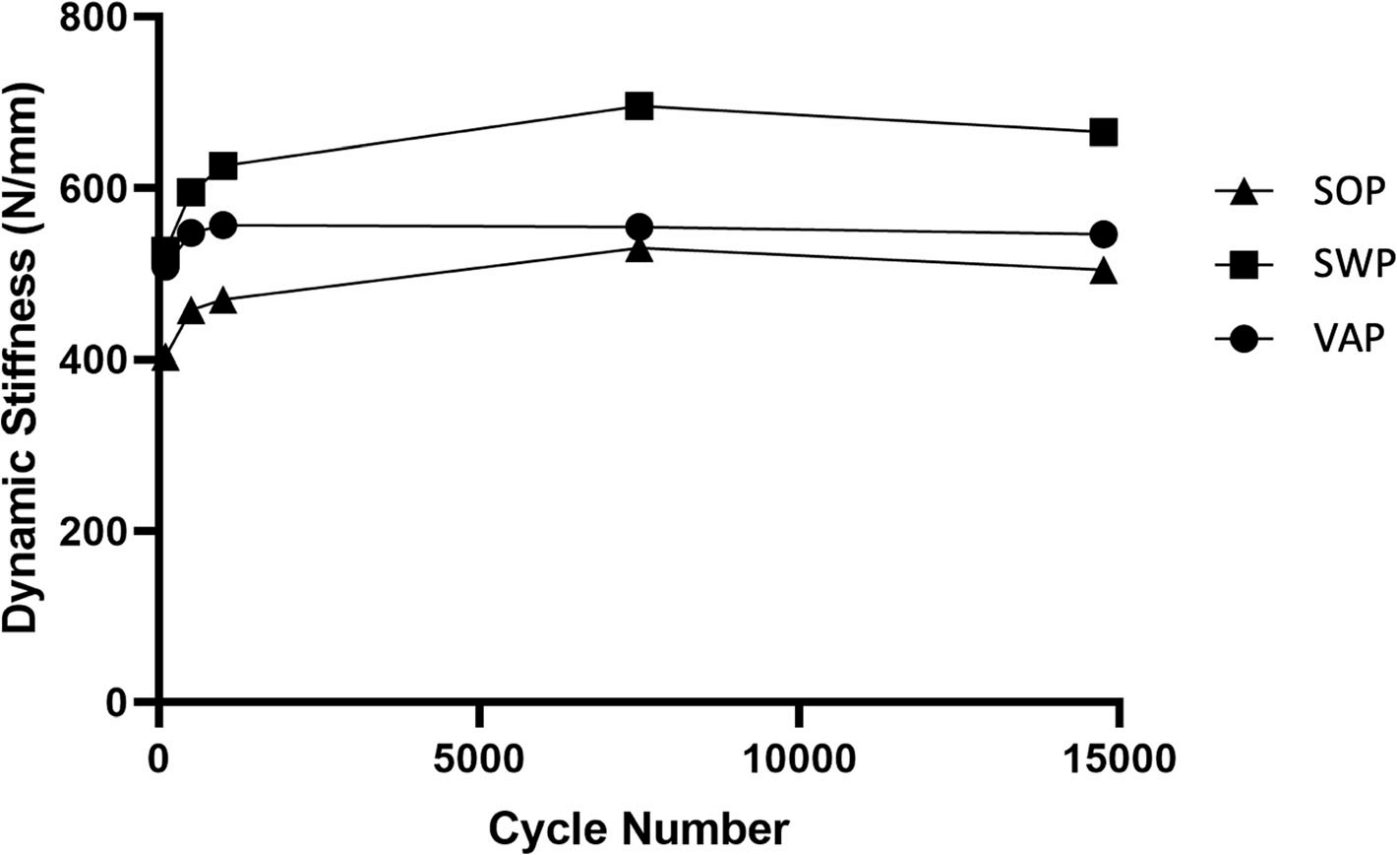
Cyclic stiffness for SOP, SWP, and VAP constructs

Total displacement (Table 1) following cyclic loading was evaluated between constructs. Weight was found to be insignificant and excluded from analysis. No significant differences were noted for cyclical displacement. Stiffness throughout cyclic testing was also evaluated at 100, 500, 1000, 7500, and 14,750 cycles. Weight was insignificant and excluded from analysis. No significant differences were noted in cyclical stiffness. The trends in cyclical displacement and stiffness between constructs are illustrated in Figs. 1 and 2.

**Table 1:** Comparison of cyclic displacement, stiffness, and comparison between SWP, SOP, and VAP fixations

Fixation	Displacement (mm)	Stiffness at 100 Cycles (N/mm)	Stiffness at 500 Cycles (N/mm)	Stiffness at 1000 Cycles (N/mm)	Stiffness at 7500 Cycles (N/mm)	Stiffness at 14750 Cycles (N/mm)
SWP	0.77	415.3	595.27	626.53	695.77	665.16
SOP	0.682	527.15	463.13	483.98	526.91	496.94
VAP	0.799	508.69	548.01	557.14	555.18	546.42
*p-value*	0.8	0.414	0.6014	0.5336	0.2732	0.3298

*SWP* screw/wire/polymethylmethacrylate, *SOP* string-of-pearls™ plate, *VAP* veterinary acetabular plate, Values reported as mean

For ultimate load and failure load, 2 samples were excluded (1 SOP sample and 1 SWP sample) due to aberrant or incorrectly recorded data via the testing machine. For failure load, the animal weight-fixation interaction was found to be significant (*P* = 0.0201) and further analysis was compared at the 25th (53.9 lbs), 50th (60.6 lbs), and 75th percentiles (69.2 lbs). Failure load data is summarized in Table 2. There was significant difference with superior failure load of SOP compared to SWP in the 75th percentile (*P* = 0.0021), and superior failure load of SOP compared to VAP in the 50th (*P* = 0.0232) and 75th percentiles (*P* = 0.0058).

**Table 2:** Comparison of ultimate load, stiffness, displacement at failure, and failure load at 25th, 50th, and 75th percentile of animal weight

Fixation		Ultimate Load (N)	Stiffness (N/mm)	Failure Load 25th Percentile (N)	Failure Load 50th Percentile (N)	Failure Load 75th Percentile (N)
SWP		1603.64 ± 170.15	344.58 ± 44.66	1720.74 ± 295.80	1513.74 ± 204.84	1248.05 ± 220.85
SOP		2182.02 ± 176.15	369.39 ± 44.66	1689.80 ± 232.64	1982.79 ± 188.53	2358.87 ± 238.50
VAP		1552.91 ± 169.94	229.56 ± 43.12	1312.38 ± 225.96	1347.27 ± 183.42	1392.06 ± 220.37
	*SOP* vs *SWP*	0.0331*	*0.8786*	*0.9937*	*0.1027*	0.0021*
*p-value*	SWP vs VAP	0.9761	0.1001	*0.4424*	*0.7458*	*0.8030*
	SOP vs VAP	0.0218*	0.0417*	*0.3179*	0.0232*	0.0058*

*SWP* screw/wire/polymethylmethacrylate, *SOP* string-of-pearls™ plate, *VAP* veterinary acetabular plate. Values reported as mean ± SEM. Significant comparisons indicated by an asterisks (*)

**Fig. 3:**
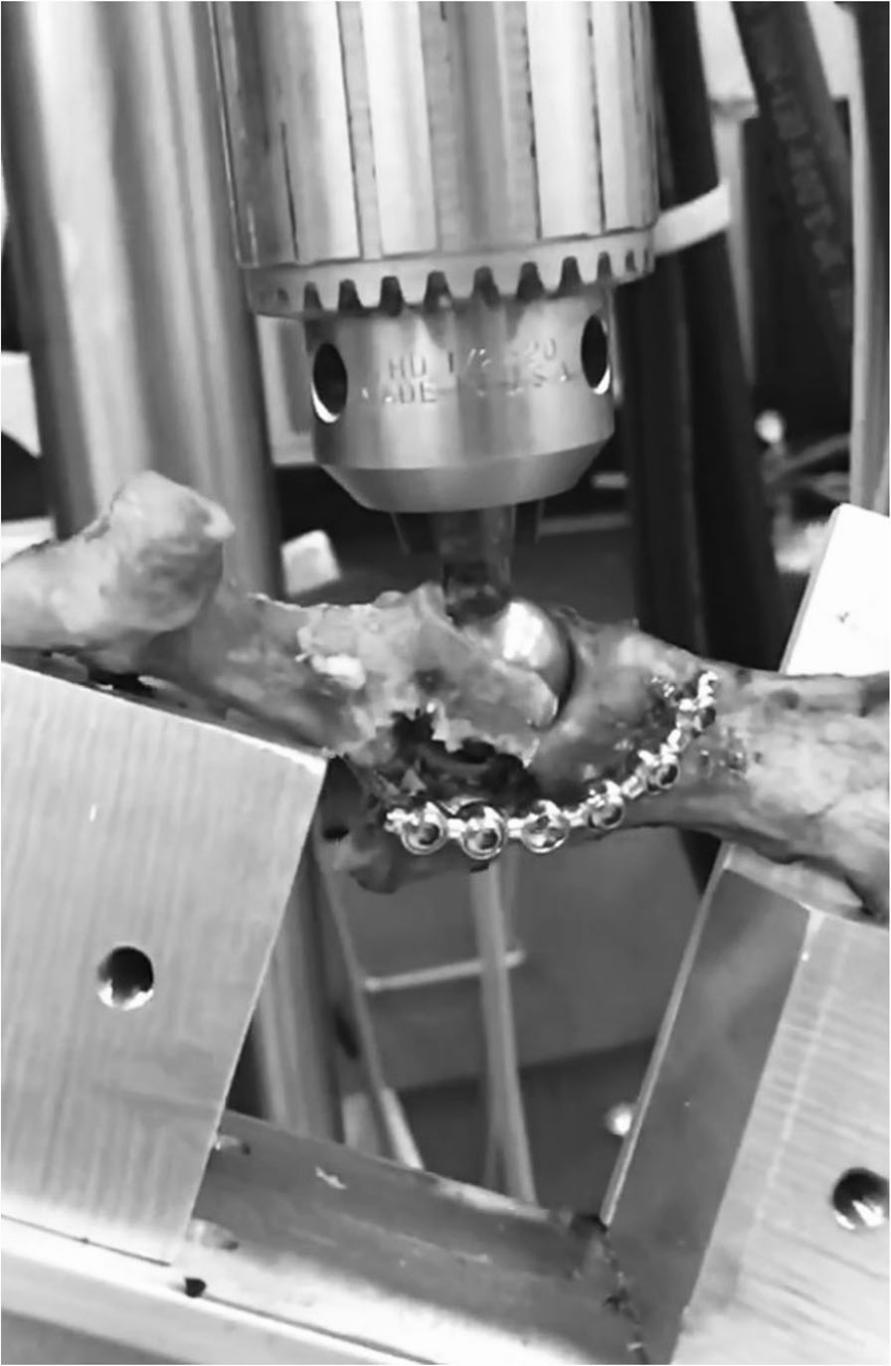
String-of-Pearls™ construct failure via caudal screw cut-out

With ultimate load and stiffness, weight was found to be insignificant and was excluded from further analysis (*P* = 0.2398). Fixation method was found to be significant in comparison between groups in stiffness (*P* = 0.038) and ultimate load (*P* = 0.0144). Data means and comparison between fixation types is outlined in Table 2. Summarily, stiffness of the SOP construct was found to be significantly greater than the VAP construct (*P* = 0.0417). There was no significant difference between the SOP construct and SWP construct (*P* = 0.8786) or between the SWP and VAP constructs (*P* = 0.1). Regarding ultimate load, SOP constructs were significantly greater than SWP (*P* = 0.0331) and VAP constructs (*P* = 0.0218). There were no significant differences between the SWP and VAP constructs (*P* = 0.9671). The modes of failure for the SOP constructs included caudal screw cut-out (5) (Fig. 3), cranial screw cut-out (3), screw deformation/breakage (2), and plate deformation (2). The VAP failed via caudal screw cut-out (7), cranial screw cut-out (1), screw deformation (1), and plate deformation (3). The SWP construct failed solely through PMMA fracture, most commonly near the caudal screws.

## Discussion

The proposed hypothesis was partially correct. There were significant differences between the SOP constructs and the SWP and VAP constructs in ultimate load and the SOP constructs were significantly different to VAP constructs in stiffness of the fixation. Similarly, SOP failure load was significantly different to both SWP and VAP constructs in the upper percentiles of animal weight as seen in Table 2. These findings may be interpreted to strengthen the case of using locking plate designs for acetabular fractures.

SOP plates are reportedly stronger in bending but weaker in torsion when compared with locking compression plates [5, 16]. SOP’s reported mechanical superiority has been attributed to the round profile of the implant which results in a higher area moment of inertia when compared to standard plates. [4, 13, 14, 17, 18]. Also, the locking plate design allows fixed angular stability as an internal fixator [19]. In regard to mode of failure, the SOP appeared to fail primarily through screw cut-out from the caudal fracture segment (Fig. 3) and is likely explained by the thinner bone in the caudal segment and limited screw purchase (two screws). The higher failure load force seen in SOP constructs in animals of higher weight is likely due to increased cortical bone thickness. Similarly, the VAP also failed primarily through caudal segment fracture and screw cut-out. Some VAP specimens also experienced plate bending/torsion that resulted in change of angle of the acetabulum and luxation of the force being applied. The SWP constructs failed with fracture of the PMMA. Despite the mechanical differences seen between constructs, the loads to failure for all groups were greater than what would be deemed normal forces applied to the post-operative patient. It is expected that all three constructs would be sufficient to maintain reduction and promote stability for healing. Therefore, ease of application and post-operative fracture alignment should also be considered.

The authors hypothesized that fracture reduction would be improved with the String-of-Pearl plating system compared to the SWP and VAP constructs as locking plates do not require accurate contouring to the degree of a standard compression plate. In a 2008 study, Amato et al. found no apparent advantage of locking plates compared to standard plating using the 2.4 mm uniLOCK reconstruction plate for repair of simulated acetabular fractures [7]. The authors hypothesized the lack of difference may be due to the ex vivo nature allowing excellent contouring of the plates that were used in the standard cortical screw groups as well as the use of interfragmentary Kirschner wires reducing malalignment for both groups [7]. Similarly, in the current study, there was no significant difference found between the three groups in regard to impression casting or acetabular diameter alteration and the ex vivo design should be considered as a possible cause. Unlike the clinical setting, the author was able to visualize the entire acetabulum and ensure alignment during fixation application. With in-vivo application, only the dorsal rim of the acetabulum is typically visualized, increasing the likelihood of error in reduction. Also, plate contouring with the VAP group was more accurate than is likely in the clinical setting as all tissue had been removed and the bone could be visualized from multiple angles during application. Decreased accuracy in plate contouring in the clinical setting may lead to loss of reduction with a compression style plate that was not seen in this study. One option previously reported [7] to improve reduction is to pre-place K-wires prior to plate application, similar to the SWP design. This could be considered with both the VAP and SOP designs to improve overall reduction. Subjectively, despite the lack in significant differences, it was easier for the author to maintain complete reduction using SWP compared to the plating techniques, possibly due to K-wire application.

The SOP construct was subjectively easier to apply when compared to the VAP and SWP constructs. Compared to the VAP, the SOP locking design allowed decreased accuracy of plate contouring prior to application. The authors believe this would be a significant advantage in the clinical setting where decreased visualization at the acetabulum is common. The SWP construct was generally easy to apply but did require finding proper locations for K-wire placement, which the SOP constructs did not require.

The cyclic testing was emulated after a previous study by Amato et al. [7] where they assumed proper confinement after surgery resulting in < 200 m/day of walking, reaching a total of 15,000 cycles for the estimated restriction period of 6–8 weeks. The lack of significance between any constructs for cyclical displacement and cyclical stiffness suggests that all three fixation techniques would provide adequate repair for expected loading after surgery. However, it was interesting to note that the SWP fixation group was stiffer, although not significant, than the plate constructs. This finding is expected given the more ductile nature of metal implants compare to PMMA. The custom-designed testing apparatus was created to allow different sizes of hemipelves to be used with ease and to remove the need of potting. During cyclic testing, it was noted that there was minor flexibility (< 1 mm displacement) in the apparatus. This was consistent between all tests and is unlikely to have any consequence on the results of the comparisons as all samples were tested using the same apparatus. Furthermore, each construct’s cyclical displacement remained unchanged after approximately 2000 cycles (Fig. 1), which likely represents settling of the construct, and each sample failed during destructive testing by failure of the implants and not the testing apparatus. The testing apparatus was straight forward to use and allowed for a prompt transition between the testing of multiple specimens. However, an alternative for future studies would be to build the testing apparatus out of stiffer material such as stainless steel.

Other limitations include uniaxial loading design and using a basic two-piece fracture model. The simplified fracture model does not account for all clinical variations but was chosen to reproduce common acetabular fracture location [7, 10]. The SWP constructs utilized polymethylmethacrylate and were tested between 2 and 18 h after application. Previous studies have shown curing time until maximal strength can take months with a significant difference between 4 weeks and 26 weeks [20]. A recent study has shown veterinary bone cement has a 7% increase in bending strength, 69% increase in hardness, and 24% increased load to fracture between 30- and 60-min post application [21]. The variable and short curing time in this cadaveric study may have played a role in total values of strength compared to the clinical scenario. However, the stiffness and loads achieved were comparable to the other constructs and well above expected in vivo forces. Furthermore, we chose a standardized volume of PMMA that was appropriate for the average size of cadaver we were testing. Clinical variation in PMMA volume and molding technique may affect the strength of the construct. Lastly, the slight variation in cadaver size may have had impact on the construct strengths. This was accounted for in the paired design of the study and animal weight was considered as a covariate in our statistical model.

## Conclusions

Although the ease of application for the SOP implant was subjectively better than other implants, no significant differences were noted with regard to fracture reduction scores in this ex-vivo model. The SOP construct did show significantly higher stiffness compared to the VAP and greater ultimate and failure load compared to the VAP and SWP fixations. This may or may not be clinically relevant as the loads reached were greater than what is expected to be seen in clinical patients. The SOP implant appears to provide suitable strength for repair of simple canine acetabular fractures. Further clinical studies investigating the use of SOP for acetabular fractures are warranted.

## Methods

This was a canine cadaveric study using 18 pelves (36 hemipelves) from animals euthanized at local human societies for reasons unrelated to this study. Using data from previous studies [3, 7], it was determined that 11 samples per group would be adequate to achieve a power of 0.80 and alpha of 0.05 (12 per group were used). Study inclusion criteria included a body weight of 20–40 kg and no evidence of orthopedic disease upon visual inspection of the coxofemoral joints following dissection. After harvest, pelves were cleaned of soft tissue, separated into hemipelves by a sagittal saw with a cut made at the pubic symphysis, wrapped in saline moistened towels, placed in plastic bags, and stored at − 20 °C for 1–2 months until testing.

### Osteotomy

**Fig. 4:**
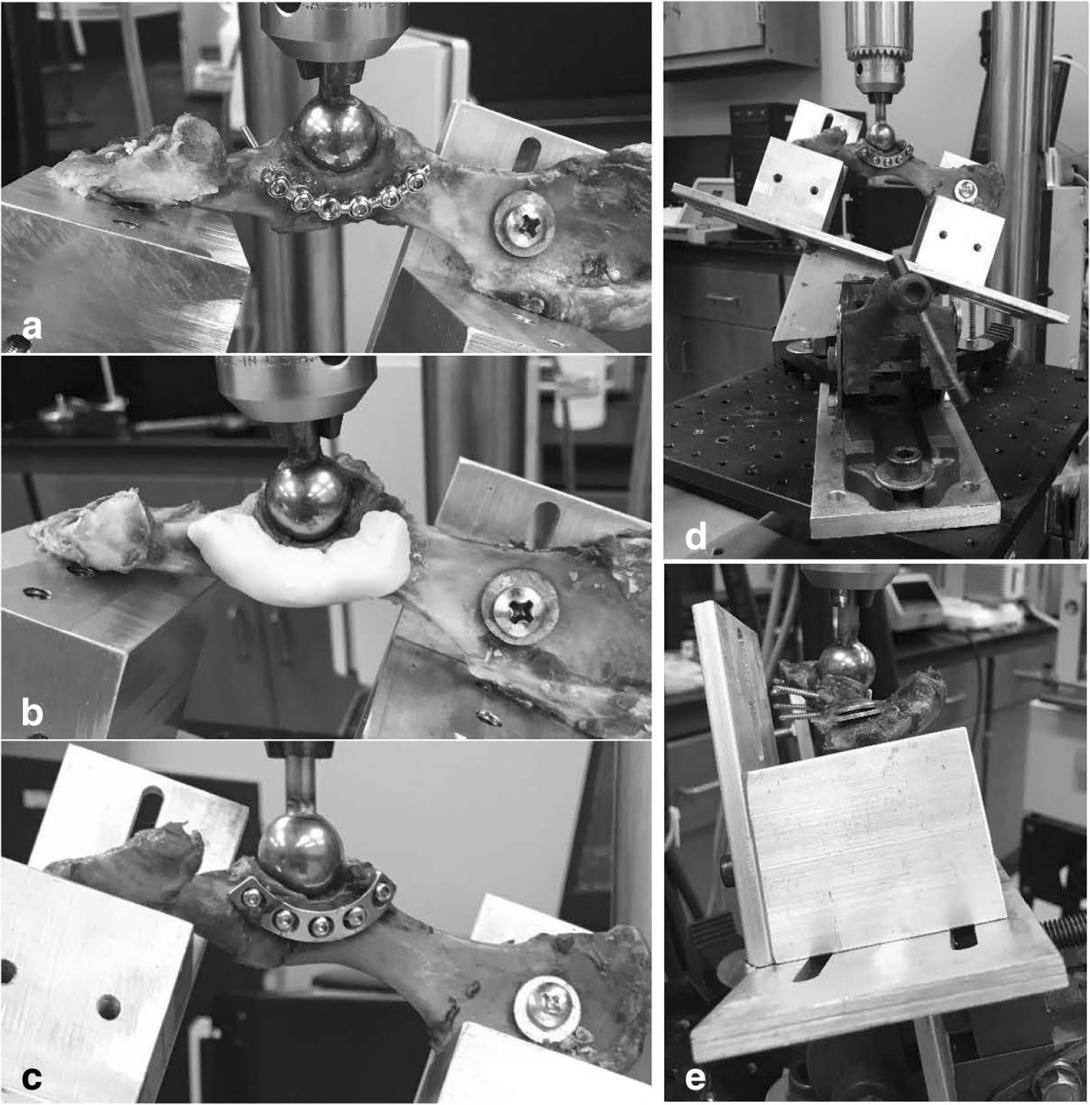
**a** String-of-Pearls™ plate contouring, application, and mounting. **b** Screw/Wire/Methylmethacrylate fixation demonstrating application and mounting. **c** Veterinary Acetabular Plate application and mounting. **d** Demonstration of the testing apparatus to replicate 110° hip flexion. **e** Demonstration of the testing apparatus to replicate 105° hip abduction

Before osteotomy creation, each hemipelvis was thawed at room temperature for 2–3 h then randomly assigned to one of three fracture fixation groups (12 hemipelves per group): 2.7 mm SOP, 2.7 mm VAP, and SWP construct. The hemipelves were stratified into groups to maintain pairing by drawing straws, resulting in 3 groups of 6 (SOP:VAP, SOP:PMMA, and VAP:PMMA). Once assigned to a group, the fixation for each hemipelvis was assigned via coin toss. Each acetabulum’s craniocaudal width was measured using a caliper and recorded before and after fixation. A transverse acetabular fracture was created at the caudal 2/3rds of the acetabulum (Fig. 4). It was started with a sagittal saw, beginning just caudal to the pubic ramus and extended dorsally through the cortical and cancellous bone without entering or damaging the acetabular surface. The fracture was then completed using an osteotome/mallet as previously described [3, 8, 11].

### Fixation

Each hemipelves was repaired by the same investigator (JAB) using previously described protocols (Fig. 4) [1, 3, 7, 8, 12, 22]. Briefly, AO point-to-point reduction forceps were used to maintain reduction and either a 5-hole 2.7 mm VAP^1^ or a 6-hole 2.7 mm SOP^2^ was applied using cortical screws. Two cortical screws^3^ were placed in the caudal segment and 3 screws in the cranial segment for both groups. The SOP group left one screw hole open above the simulated fracture to better represent the typical clinical scenario. The plates were contoured to the dorsal acetabular rim prior to screw placement and then placed in standard ASIF technique and in accordance with manufacturer recommendations for the SOP group [17]. For the SWP group, 0.062 interfragmentary K-wires^4^ were placed craniodorsally to caudoventrally and caudodorsally to cranioventrally, followed by bi-cortical 2.7 mm screw placement on either side of the osteotomy approximately 7 mm from the osteotomy with the screws inserted to a point that the screw head was approximately 3 mm proud of the cis-cortex. The construct was connected using 18-gauge orthopedic wire^5^ in a figure-of-eight pattern around the protruding portions of the screws. The orthopedic wire was tightened by hand above the dorsolateral surface of the acetabulum. PMMA^6^ was mixed to putty consistency, conformed to 1 cm × 2 cm × 1 cm, pressed over the implants and allowed to cure.

### Impression casting

Impression casting and gross evaluation was performed prior to biomechanical testing to assess articular alignment. Type 0 very high consistency polyvinylsiloxane impression material^7^ was applied to the articular surface of the acetabula to create a cast of the articular surface [11]. This was evaluated for a step defects or other incongruencies of the acetabular surface by a blinded observer (JNA). Each hemipelvis was assigned to a grade of reduction as previously described [11]. Grade I indicated no evident flaw or malalignment to the articular surface. Grade II consisted of any acetabulum with minor malalignment or gap < 1 mm. Grade III referred to acetabula with poor reduction including obvious malalignment or a gap > 1 mm.

### Biomechanical testing

Prior to biomechanical testing, each hemipelvis was secured in a custom-made apparatus (Fig. 4) in an inverted position and an orientation relative to the force actuator to simulate normal standing angles of 110° hip flexion and 105° hip abduction [7, 14, 23]. This apparatus was designed from aluminum with placement of 1 screw within the ischial table and 1 screw in the wing of the ilium. In all specimens, it was ensured that there was solid contact between the ischium and dorsal ilium to the “table” of the testing apparatus to maintain consistency. Likewise, the previously described desired angles were ensured prior to each test. To approximate clinical loading of the acetabulum, the fixation groups were tested in three-point bending using a material testing machine^8^ via sinusoidal loading of the acetabulum with a spherical testing apparatus^9^ (19 mm or 23 mm depending on the size of acetabulum). Specimens were loaded for 15,000 cycles between previously described levels of 22 and 245 N at 2 Hz to simulate expected workload during post-operative confinement [7]. This was followed by single load to failure with initial loading of 20 N and displacement rate of 1 mm/s [3, 7, 8, 11].

### Data analyses

For the cyclical testing, peak and valley data were collected from the material testing machine and used to determine displacement. Stiffness was measured at 100, 500, 1000, 7500, and 14,750 cycles and was defined as the slope of the linear portion of the force versus displacement curve at the given cycle. For the destructive testing, ultimate load, stiffness, and failure load were calculated from force versus displacement curves using standard statistical spreadsheet software^10^. Stiffness (N/mm) was defined as the slope of the elastic portion of the force versus displacement curves. Ultimate load was defined as the maximum load obtained prior to catastrophic failure of the constructs. Failure load was defined as yield (plastic deformation) determined from the force versus displacement curves via a 2 mm displacement offset method. In samples with no appreciable plastic deformation prior to catastrophic failure, ultimate load was used as the failure load value for statistical comparisons. Samples were video recorded during testing and inspected post-failure to determine mode of failure.

### Statistical methods

The effect of fixation method on cyclical displacement, cyclical stiffness, stiffness, ultimate load, failure load, and the magnitude of difference between the acetabular diameters before and after fixation were assessed using linear mixed models with PROC MIXED in SAS^11^. Separate mixed models were fit for the six outcomes. Initial models included fixation method, body weight, and their interaction as fixed effects with dog identity as a random effect. If the interaction term was not significant, it was removed and the model refit. Similarly, if body weight did not have a significant effect, it was also removed. The distribution of residuals was evaluated for each model to ensure the assumptions of normality and homoscedasticity had been met. Mixed model ordinal logistic regression using PROC GLIMMIX with the Laplace estimation method in SAS for Windows was used to determine if there was an association between grade and fixation method. As above, the initial model included fixation method, body weight, and their interaction as fixed effects with dog identity as a random effect. If the interaction term was not significant, it was removed and the model refit. Similarly, if body weight did not have a significant effect, it was also removed. An alpha level of 0.05 was used to determine statistical significance for all models.

## Data Availability

The datasets used and/or analyzed in the current study are available from the corresponding author on reasonable request.

## Abbreviations

PMMA: Polymethylmethacrylate
SOP: String-of-Pearls™
SWP: Screw/Wire/Polymethylmethacrylate
VAP: Veterinary acetabular plate

## Footnotes

1 Veterinary Acetabular Plate (VAP): Orthomed, Halifax, West Yorkshire, UK

2 String-of-Pearls™ (SOP): Orthomed, Halifax, West Yorkshire, UK

3 2.7 mm cortical screw: Orthomed, Halifax, West Yorkshire, UK

4 Kirschner wire: Orthomed, Halifax, West Yorkshire, UK

5 316 stainless steel orthopedic wire: Orthomed, Halifax, West Yorkshire, UK

6 Veterinary Bone Cement: BioMedtrix, Whippany, NJ, USA

7 Extrude XP Putty: Kerr Dental, Orange, CA, USA

8 MTI-2 K Universal Testing Machine: Measurements Technology, Inc., Marietta, GA, USA

9 316 Stainless Steel Precision Ball: McMaster-Carr, Douglasville, GA, USA

10 GraphPad Prism 8: GraphPad Software, Inc., La Jolla, CA, USA

11 SAS for Windows v9.4: SAS Institute Inc., Cary, NC, USA.
